# Case Report: Disruption of Resting-State Networks and Cognitive Deficits After Whole Brain Irradiation for Singular Brain Metastasis

**DOI:** 10.3389/fnins.2021.738708

**Published:** 2021-10-27

**Authors:** Martin Kocher, Christiane Jockwitz, Christoph Lerche, Michael Sabel, Philipp Lohmann, Gabriele Stoffels, Christian Filss, Felix M. Mottaghy, Maximilian I. Ruge, Gereon R. Fink, Nadim J. Shah, Norbert Galldiks, Svenja Caspers, Karl-Josef Langen

**Affiliations:** ^1^Institute of Neuroscience and Medicine (INM-4), Research Center Juelich, Juelich, Germany; ^2^Department of Stereotaxy and Functional Neurosurgery, Center for Neurosurgery, Faculty of Medicine and University Hospital Cologne, Cologne, Germany; ^3^Center of Integrated Oncology, Universities of Aachen, Bonn, Cologne and Duesseldorf, Cologne, Germany; ^4^Institute of Neuroscience and Medicine (INM-1), Research Center Juelich, Juelich, Germany; ^5^Institute for Anatomy I, Medical Faculty and University Hospital Düsseldorf, Heinrich Heine University Duesseldorf, Duesseldorf, Germany; ^6^Department of Neurosurgery, Medical Faculty, Center of Neuro-Oncology, Heinrich-Heine-University Düsseldorf, Düsseldorf, Germany; ^7^Department of Nuclear Medicine, University Hospital Aachen, Rheinisch-Westfaelische Technische Hochschule (RWTH) Aachen University, Aachen, Germany; ^8^Department of Radiology and Nuclear Medicine, Maastricht University Medical Center, Maastricht, Netherlands; ^9^Institute of Neuroscience and Medicine (INM-3), Research Center Juelich, Juelich, Germany; ^10^Department of Neurology, Faculty of Medicine and University Hospital Cologne, University of Cologne, Cologne, Germany; ^11^Department of Neurology, University Hospital Aachen, Rheinisch-Westfaelische Technische Hochschule (RWTH) Aachen University, Aachen, Germany; ^12^Juelich-Aachen Research Alliance—Section JARA-Brain, Juelich, Germany

**Keywords:** brain metastases, whole-brain radiotherapy, functional magnetic resonance imaging, cognitive function, case report

## Abstract

**Introduction:** Long-term survivors of whole brain radiation (WBRT) are at significant risk for developing cognitive deficits, but knowledge about the underlying pathophysiological mechanisms is limited. Therefore, we here report a rare case with a singular brain metastasis treated by resection and WBRT that survived for more than 10 years where we investigated the integrity of brain networks using resting-state functional MRI.

**Methods:** A female patient with a left frontal non-small cell lung cancer (NSCLC) brain metastasis had resection and postoperative WBRT (30.0 in 3.0 Gy fractions) and stayed free from brain metastasis recurrence for a follow-up period of 11 years. Structural magnetic resonance imaging (MRI) and amino acid [O-(2-[^18^F]fluoroethyl)-L-tyrosine] positron emission tomography (FET PET) were repeatedly acquired. At the last follow up, neurocognitive functions and resting-state functional connectivity (RSFC) using resting-state fMRI were assessed. Within-network and inter-network connectivity of seven resting-state networks were computed from a connectivity matrix. All measures were compared to a matched group of 10 female healthy subjects.

**Results:** At the 11-year follow-up, T2/FLAIR MR images of the patient showed extended regions of hyper-intensities covering mainly the white mater of the bilateral dorsal frontal and parietal lobes while sparing most of the temporal lobes. Compared to the healthy subjects, the patient performed significantly worse in all cognitive domains that included executive functions, attention and processing speed, while verbal working memory, verbal episodic memory, and visual working memory were left mostly unaffected. The connectivity matrix showed a heavily disturbed pattern with a widely distributed, scattered loss of RSFC. The within-network RSFC revealed a significant loss of connectivity within all seven networks where the dorsal attention and fronto-parietal control networks were affected most severely. The inter-network RSFC was significantly reduced for the visual, somato-motor, and dorsal and ventral attention networks.

**Conclusion:** As demonstrated here in a patient with a metastatic NSCLC and long-term survival, WBRT may lead to extended white matter damage and cause severe disruption of the RSFC in multiple resting state networks. In consequence, executive functioning which is assumed to depend on the interaction of several networks may be severely impaired following WBRT apart from the well-recognized deficits in memory function.

## Introduction

In patients with singular brain metastases of primary solid tumors, neurosurgical resection followed by postoperative whole brain radiation therapy (WBRT) was a common therapeutic approach in the recent decades (Patchell et al., 1998; Gaspar et al., 2010). While WBRT increases local control at the resection site and reduces the number of newly occurring remote brain metastases (Patchell et al., 1998; Kocher et al., 2011; Brown et al., 2017), evidence is increasing that it may cause progressive cognitive deficits and impairment of quality of life especially in long-term survivors of the underlying metastatic cancer disease (Lin et al., 2013; Soffietti et al., 2013, 2017; Pinkham et al., 2015; Brown et al., 2017). Cognitive deficits following WBRT have been mainly observed in the domain of learning and memory function and are assumed to be caused by radiation-induced damage to the bilateral hippocampus (Chang et al., 2009), an insight that had led to the concept of hippocampal-sparing WBRT (Gondi et al., 2014; Brown et al., 2020; Giuseppe et al., 2020).

However, modern concepts of brain function suggest that cognitive performance in different domains does not rely solely on the function of specialized single cortical regions but rather depends on the interaction of multiple cortical regions organized in distributed functional networks. Several of these functional networks have been identified using resting-state functional MRI (rs-fMRI), and a set of cognitive functions have been assigned to them (Yeo et al., 2011; Schaefer et al., 2018). While alterations in whole-brain resting-state functional connectivity (RSFC) induced by chemotherapy of breast cancer patients (“chemo brain”) have been studied to some extent (Bruno et al., 2012; Kesler et al., 2013; Piccirillo et al., 2015; Tao et al., 2017, 2020), reports regarding the effect of WBRT on RSFC are rare (Mitchell et al., 2020). Because WBRT, as chemotherapy, potentially affects multiple cortical regions and networks, it can be hypothesized that cognitive deficits after WBRT affect functional connectivity both within and between multiple networks and may result in deficits in multiple cognitive domains.

Therefore, we here took the rare opportunity to assess resting-state functional connectivity and cognitive performance in a long-term survivor of brain metastasis treated with WBRT who participated in a prospective study. The patient studied here suffered from a synchronous singular brain metastasis of a non-small cell cancer (NSCLC) and received resection and WBRT for the brain metastasis and resection and adjuvant chemotherapy for the primary tumor. The long survival and follow-up period of more than 10 years is rarely observed and put her at risk of developing the full extent of radiation-induced brain damage and associated cognitive deficits (Kocher et al., 2011; Brown et al., 2017). For comparison, we used RSFC and cognitive performance data from a closely matched group of healthy subjects from a population-based cohort.

## Case Description

In 2008, a 48-year old, left-handed female patient trained as a physician's assistant experienced a generalized seizure caused by a left frontal brain metastasis of a NSCLC (adenocarcinoma) of the lung. The metastasis was treated by neurosurgical resection and postoperative WBRT with a total dose of 30.0 Gy in 10 daily fractions of 3.0 Gy. After completion, the primary tumor was treated by upper lobe resection and mediastinal lymphonodectomy. Due to the involvement of mediastinal lymph nodes, four courses of adjuvant chemotherapy (cis-platin/ vinorelbine) were applied and a complete remission at the intracranial and extracranial tumor sites could be achieved. During an 11-year follow up period, MR and FET PET brain imaging was conducted repeatedly. At the last follow up at the patient's age of 59.9 years, resting-state functional MRI (rs-fMRI) and cognitive testing were additionally performed. The timeline of the therapeutic interventions, diagnostics and outcomes is depicted in Table 1.

**Table 1:** Timeline of diagnostics and therapeutic interventions.

**Time (months from initial diagnosis)**	**Diagnostics/interventions**	**Outcome**
0	Generalized seizure, structural brain MRI, and FET-PET	Left parietal, contrast-enhancing, and FET-PET-positive lesion
1	Resection of lesion	Singular metastasis, histopathological type: adenocarcinoma
2–3	Whole brain irradiation, 30 Gy in 10 fractions	Well tolerated
4	Right upper lobectomy for resection of primary tumor, mediastinal lymphadenectomy	NSCLC, involvement of mediastinal lymph nodes, Stage IV
6–9	Adjuvant chemotherapy, four courses of cisplatin/ vinorelbine	Complete remission of primary tumor
10–132	Follow-up including repeated chest CT and structural brain MRI and FET-PET, no further oncologic interventions	Complete remission of primary tumor and brain metastasis
132	Last follow-up, structural brain MRI, resting-state fMRI, and FET-PET	Development of bilateral white matter lesions

## Diagnostic Assessment

### Structural MR and PET Imaging

During the follow up period of the patient, structural MR and O-(2-[18F]fluoroethyl)-L-tyrosine (FET) PET images were obtained using a high-resolution 3T hybrid PET/MR scanner equipped with a PET insert and a birdcage-like quadrature transmit head coil and an 8-channel receive coil (Siemens Tim-TRIO/ BrainPET, Siemens Medical Systems, Erlangen, Germany) (Herzog et al., 2011). MR imaging included a 3D T1-weighted magnetization-prepared rapid acquisition gradient-echo (MPRAGE) anatomical scan (176 sagittal slices, TR = 2,250 ms, TE = 3.03 ms, FoV = 256 ×256 mm^2^, flip angle = 9°, voxel-size = 1 ×1 ×1 mm^3^), a contrast-enhanced T1-weighted image (T1-CE) obtained from a second MPRAGE scan following the injection of gadolinium (0.1 mmol/kg, Dotarem^R^, Guerbet GmbH, Sulzbach, Germany), and T2-weighted (T2-SPACE, 176 slices, TR = 3.2 s, TE = 417 ms, FoV = 256 ×256 mm^2^, voxel-size = 1 ×1 ×1 mm^3^) and fluid-attenuated inversion recovery structural images (FLAIR, 25 slices, TR = 9,000 ms, TE = 3.86 ms, FoV = 220 ×220 mm^2^, flip angle = 150°, voxel-size = 0.9 ×0.9 ×4 mm^3^).

### Resting-State Functional MR Imaging

For rs-fMRI assessment, 300 functional volumes were acquired within 11 min using a gradient-echo echo planar imaging (GE-EPI) pulse sequence (36 axial slices, slice thickness 3.1 mm, repetition time TR = 2,200 ms, echo time TE = 30 ms, flip angle = 90°, FoV = 200 ×200 mm^2^, in-plane voxel-size 3.1 ×3.1 mm^2^). During image acquisition, the patient and the healthy subjects were instructed to relax and let their mind wander, but not to fall asleep. All MR protocols were exactly reproduced from a stand-alone 3T Siemens Tim-TRIO MR scanner used in the control group of the 1000BRAINS study (Caspers et al., 2014), and transferred to the hybrid scanner.

### Determination of Resting-State Functional Connectivity

Functional images were subjected to the standard pre-processing steps of the SPM12/CONN toolbox (Whitfield-Gabrieli and Nieto-Castanon, 2012) comprising motion correction with removal of outliers and CSF/ white matter signals, slice timing correction, smoothing with 5 mm FWHM, bandpass-filtering to 0.008–0.09 Hz and denoising. All structural and functional images were non-rigidly co-registered to the MNI-152 standard brain template by means of the SPM12/CONN unified segmentation/registration algorithm. For determination of RSFC, a recently developed cortical parcellation (Schaefer et al., 2018) comprising 2 ×50 regions at the lowest resolution belonging to seven resting-state networks (visual, somato-motor, dorsal attention, ventral attention, limbic, fronto-parietal control, and default mode) was imported into the CONN toolbox and used to compute full connectivity matrices from the *z*-transformed Pearson correlation coefficients between the respective nodes. From these *z*-values, the within-network connectivity (sum of positive z-values for connections in the network/total number of nodes in the network) and inter-network connectivity (sum of positive *z*-values for connections to other networks/total number of connections to other networks) was computed for each of the seven networks (Stumme et al., 2020).

### Cognitive Assessment

At the last follow up 11 years after initiation of therapy, the patient underwent a selection of neurocognitive tests using a subset of a test battery from the 1000BRAINS study (Caspers et al., 2014) that could be completed within 25–30 min and comprised the domains processing speed, attention, executive function, concept shifting, semantic word fluency, language processing, verbal working memory, visuo-spatial working memory and verbal episodic memory (Morris et al., 1989; Kalbe et al., 2004; Tombaugh, 2004).

### Matched Cohort of Healthy Subjects

As pre-therapeutic cognitive data were not available for the patient, a reference group of healthy subjects was constructed from a population-based cohort study comprising over 1,300 patients. The cohort study investigated environmental and genetic influences on the inter-individual variability of brain structure, function, and connectivity in the aging brain [1000BRAINS study (Caspers et al., 2014)]. Due to a large variability of the factors gender, age and education in this large cohort which are known to impact both on cognitive performance and network measures (Jockwitz et al., 2017; Stumme et al., 2020; Jockwitz and Caspers, 2021), we aimed at selecting a subsample that resembled the patient as close as possible. For this purpose, only women that had the exactly the same level of education (tertiary, non-academic education, ISCED level 5B, 1997 ISCED scoring system, http://uis.unesco.org/sites/default/files/documents/international-standard-classification-of-education-1997-en_0.pdf, accessed at 1.2.2018) and were of nearly the same age (±2 years) as the patient (59.9 years) were chosen from the entire cohort. This resulted resulting in a small cohort of 10 of healthy women with a mean age of 60.1 years (range 58.6–61.8 years). For comparison of cognitive test scores and within- and inter-network connectivity measures between the patient and the matched cohort, the (two-sided) one-sample *t*-test was applied (Kalpić et al., 2011). By applying this test, the observed value for the patient was assumed to represent the hypothetical mean of a patient population, and the probability distribution of the mean for the healthy cohort is compared to this value. *P* < 0.05 were regarded as significant.

## Outcome

### Clinical and Imaging Findings

During a follow-up period of 11 years, the patient stayed free from local tumor recurrence in the resection cavity, distant brain recurrence and further extracranial metastases. At the last follow up, she was in good general condition (ECOG performance score 0) and free from epileptic seizures and neurological deficits apart from a slight amnestic aphasia. She was still employed as a physician's assistant and reported to suffer from a significant reduction of working speed.

**Figure 1:**
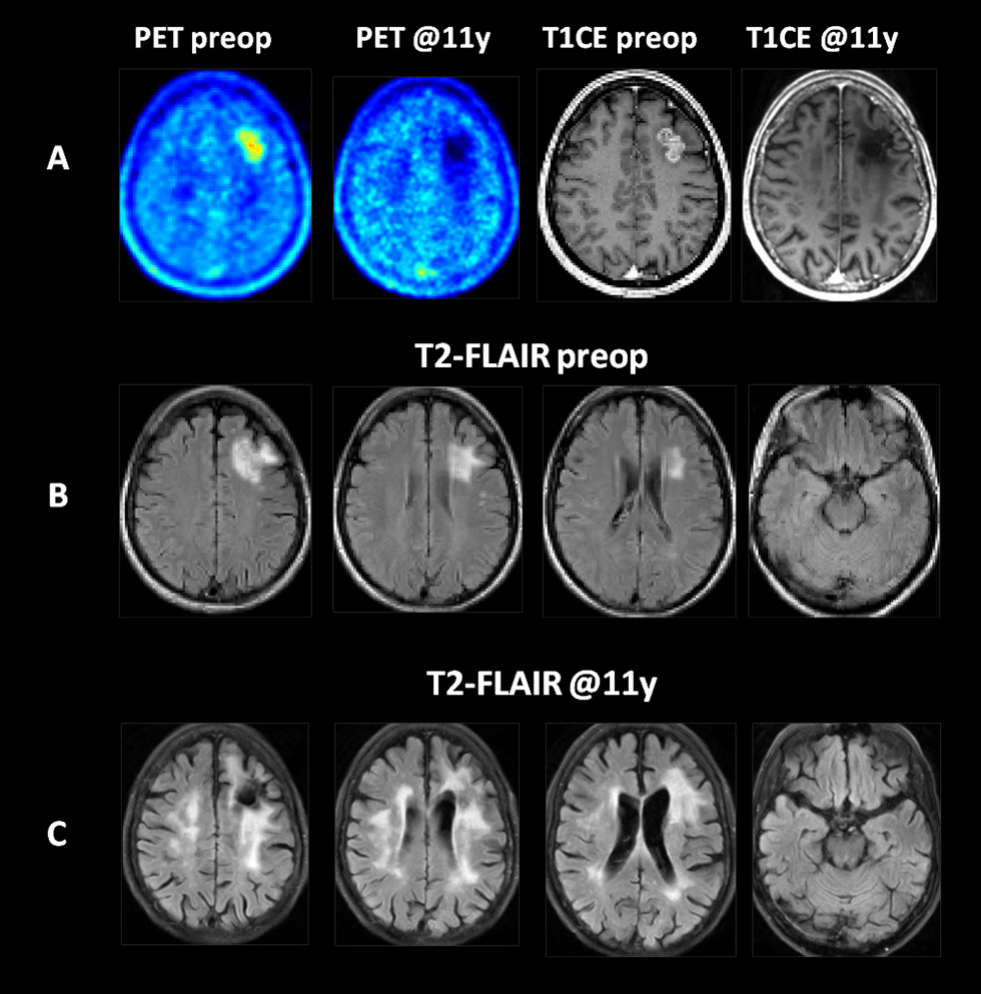
Imaging findings at initial diagnosis and at 11 years of follow-up in a patient with a left frontal NSCLC (adenocarcinoma) brain metastasis treated by resection and whole brain radiotherapy. **(A)** The T1-weighted contrast enhanced MR (T1CE) and FET PET (PET) images revealed a contrast enhancing, PET-positive metastatic tumor with no evidence of recurrence in the brain or resection cavity during follow up. **(B)** The T2-FLAIR images at initial diagnosis. **(C)** At follow-up, extensive areas of T2-FLAIR hyperintensities developed in the white matter of the bilateral dorsal frontal and parietal lobes. All images are shown after elastic registration to the MNI-152 standard brain template.

At initial diagnosis, the brain metastasis presented with localized MR contrast enhancement, increased uptake of FET and a well circumscribed, local T2/FLAIR hyperintensity covering the tumor and a small rim of normal brain tissue (Figure 1). At the last follow-up 11 years after brain metastasis resection and WBRT, the T1-weighted contrast enhanced MR and FET PET images showed no evidence of tumor recurrence, but in the T2/FLAIR MR images, extended regions of hyperintensities covering mainly the white mater of the bilateral dorsal frontal and parietal lobes while sparing most of the temporal lobes were found.

### Cognitive Test Results

**Figure 2:**
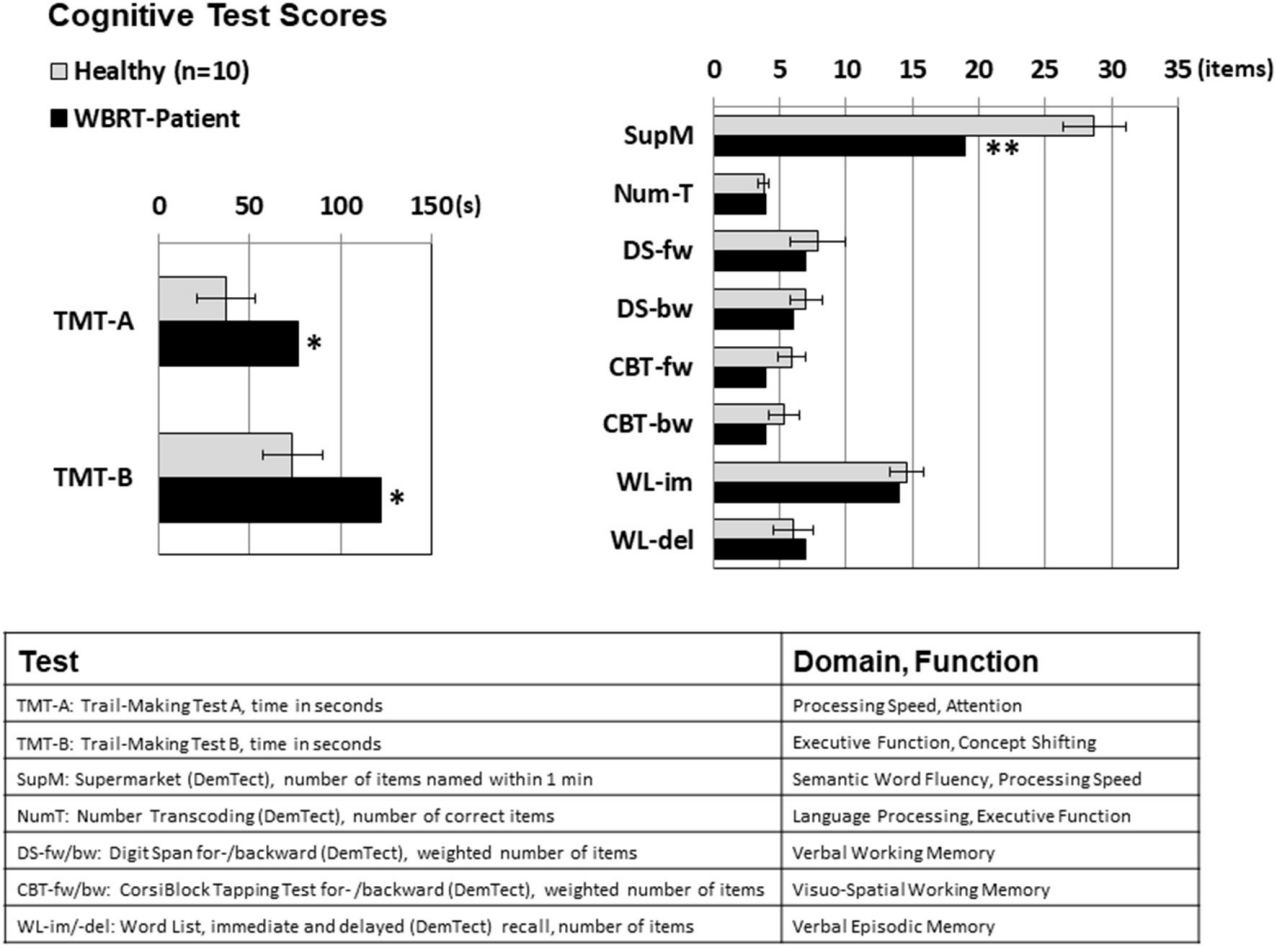
Cognitive test scores for Trail Making Tests A and B (measured in seconds, lower numbers mean better results) and for the tests scored by the number of items achieved (higher scores mean better results). Significant performance differences between the WBRT patient and the healthy subjects were observed for cognitive domains with a component of executive functioning (TMT-A: attention/ processing speed, TMT-B: executive function/concept shifting, supermarket test: semantic word fluency), while the scores for the memory domains were not significantly lowered. For the healthy subjects, means ± standard deviations are depicted; *p < 0.05, **p < 0.01, one-sample *t*-test.

Compared to the healthy subjects, the patient performed significantly worse in all cognitive domains that included executive functions, attention and processing speed (TMT-A 76 vs. 37 s, TMT-B 122 vs. 74 s, semantic word fluency test 19 vs. 29 items within 1 min), while most of the memory functions (verbal working memory, verbal episodic memory, and visual working memory) were left mostly unaffected, see Figure 2.

### Resting-State Functional Connectivity

**Figure 3:**
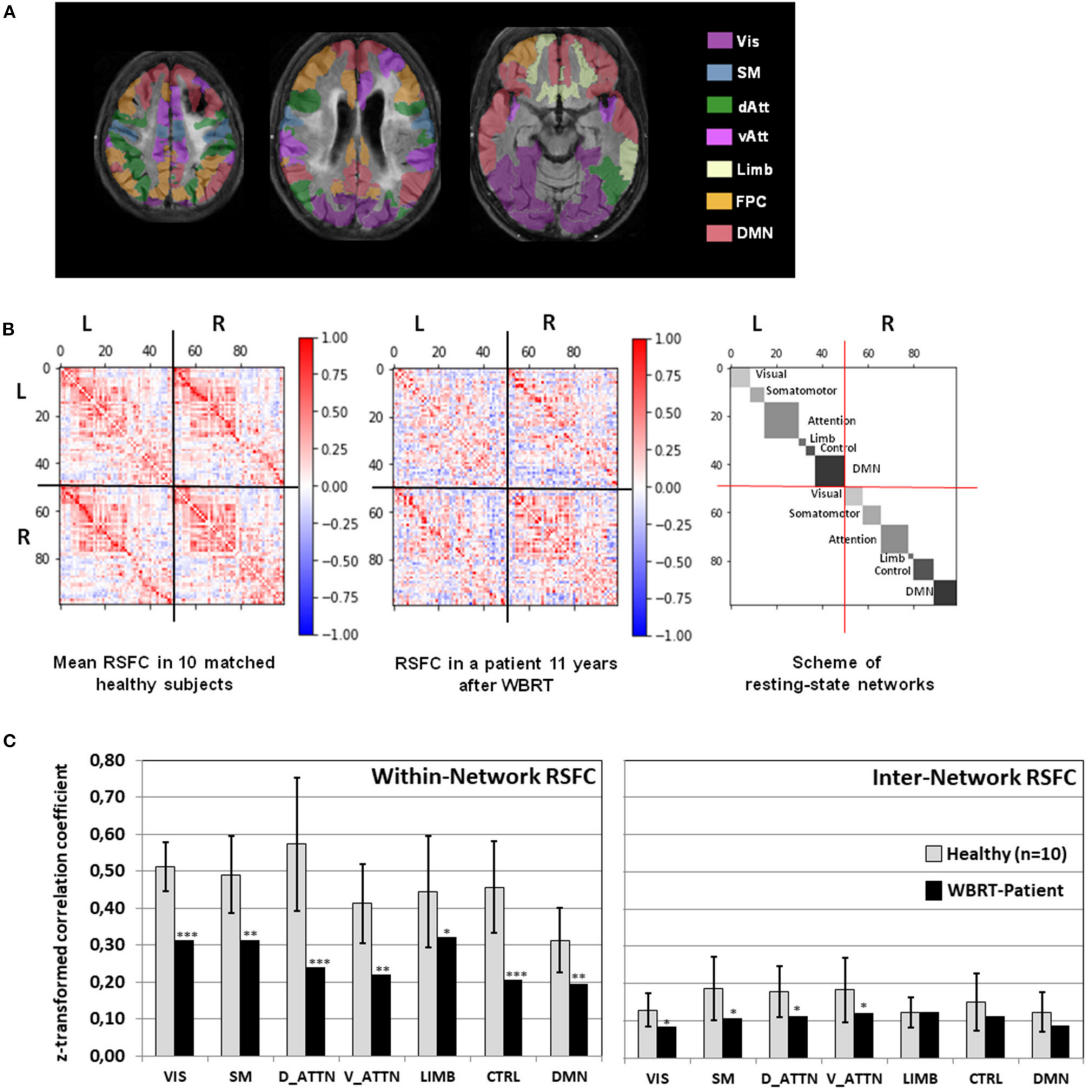
**(A)** Overlay of the patient's FLAIR images registered to the MNI-152 space with the color-coded cortical areas belonging to 7 resting-state networks. **(B)** Mean resting-state functional connectivity in 10 healthy subjects and in the patient depicted together with the scheme of resting-state networks. **(C)** Within-network and inter-network resting state connectivity (RSFC, mean *z*-normalized Pearson correlation coefficient of positive-valued connections) for each of the seven resting-state networks in the whole brain radiotherapy (WBRT) patient compared to the healthy subjects. For the healthy subjects, means ± standard deviations are depicted. *p < 0.05, **p < 0.01, ***p < 0.001, one-sample *t*-test. Vis, Visual Network; SM, Somato-Motor Network; dAtt, Dorsal Attention Network; vAtt, Ventral Attention Network; Limb, Limbic Network; FPC, Fronto-Parietal Control Network; DMN, Default Mode Network.

As depicted in Figures 3A,B, the mean RSFC matrix of the healthy subjects showed a clear pattern reflecting the structure of the underlying resting state networks. In contrast, the connectivity matrix of the patient showed a heavily disturbed pattern with a widely distributed, scattered loss of RSFC. The analysis of the within-network RSFC (Figure 3C) revealed a significant reduced connectivity within all seven networks. The dorsal attention and fronto-parietal control networks were the most severely affected and showed a reduction to <50% of the values in the healthy controls. As implied from the network structure of the brain, the inter-network RSFC was generally lower than the within-network connectivity in the healthy subjects, and was significantly further reduced in the patient for the visual, somato-motor, dorsal attention, and ventral attention networks.

## Discussion

In the present study, a NSCLC long-term survivor with metastatic brain disease who had WBRT developed extended white matter damage expressed by T2/FLAIR hyperintensities mainly covering the dorsal bilateral frontal and parietal lobes. Cognitive deficits were dominant in domains of executive functions, attention and processing speed but largely spared memory functions. The within-network connectivity of all 7 major resting state networks was significantly reduced, but the dorsal attention and fronto-parietal networks were most severely affected. A minor reduction of the inter-network connectivity was observed for the visual, somatomotor and attention networks.

### Cognitive Deficits Following WBRT

The best evidence for the typical pattern of cognitive deficits induced by prophylactic or therapeutic WBRT was obtained from randomized clinical trials where appropriate neurocognitive test sets have been applied (Tallet et al., 2012; McDuff et al., 2013). Recent trials found that, as in the present patient, not only memory, but also executive functioning is impaired as early as 3–6 months after WBRT (Brown et al., 2016, 2017). This view is also supported by a randomized trial on hippocampal-sparing WBRT where about 30% of the patients had memory deficits and a deterioration in the Trail-Making tests A (processing speed/attention) and B (concept shifting/executive function) at 6 months after WBRT (Brown et al., 2020).

Much less is known about the cognitive deficits developing after a follow-up time of 12 months or more after WBRT because only a small portion of patients survive this period, but it appears that verbal memory, executive functioning and processing speed are mostly affected (McDuff et al., 2013). Also, it is commonly assumed that injury to the white matter represents the major cause of cognitive deficits after WBRT and may progress beyond this time point (Greene-Schloesser et al., 2012). As in the case presented here, typical white matter changes detected by FLAIR MR imaging are dominantly located in the periventricular deep white matter and in the centrum semiovale and cover the dorsal frontal and parietal rather than the temporal lobes harboring the hippocampus (Fujii et al., 2006; Sabsevitz et al., 2013; Bovi et al., 2019). Therefore, it can be hypothesized that memory deficits are transient effects potentially caused by depletion of neuronal stem cells of the hippocampus (Peissner et al., 1999), whereas late cognitive deficits develop as a consequence of white matter injury to more distributed dorsal brain regions (Makale et al., 2017).

### Functional Brain Connectivity Changes After WBRT

Regarding the changes in functional brain connectivity as determined by fMRI and induced by WBRT, to the best of our knowledge, only one other single case has been reported so far (Mitchell et al., 2020). This other reported patient also developed a decline in multiple cognitive domains including executive functions/visuo-constructional skills, naming, attention/calculations, memory and orientation 12 months after WBRT. The RSFC analysis was carried out 9 months after WBRT and revealed that most functional networks became aberrant where the medial temporal lobe network and the fronto-parietal network were affected most.

In the patient of the present study, the within-network connectivity was primarily affected, a finding that has also been made in the aging brain (Stumme et al., 2020). Importantly, the dorsal attention network which is responsible for goal-directed, voluntary control of visuospatial attention (Corbetta and Shulman, 2002; Kincade et al., 2005; Vossel et al., 2012, 2014) and the fronto-parietal network which is involved in sustained attention, complex problem-solving and working memory (Menon, 2011) were affected most. This observation fits to the other results obtained here in two ways. First, these networks are located in brain regions where the white matter was affected most. Second, the scores for cognitive tests that included a dominant component of executive functioning, directed attention and processing speed (TMT-A, TMT-B, and test verbal fluency test) were reduced strongest in the patient.

The slight reduction of inter-network connectivity after WBRT is probably also a consequence of radiation damage to the white matter. However, due to the network structure of the brain, inter-network connectivity can be expected to be lower than within-network connectivity, and has been found to show a compensatory increase as a response to diminishing within-network connectivity in older adults (Jockwitz and Caspers, 2021). Thus, a possible explanation for the pattern observed here at long time after WBRT may reflect the ability of the brain to partly compensate the irradiation-induced damage by functional reorganization.

### Patients Perspective

At the last follow up, the patient was still employed as a physician's assistant and reported to live a satisfactory life. However, she claimed that performance speed for daily accomplishments at home and working tasks was significantly reduced which had caused some negative remarks from her colleagues. She did not regret her decision to have whole-brain radiation because, in her opinion, it had prevented the development of new brain metastases.

## Conclusion

As demonstrated here in a patient with a metastatic NSCLC and long-term survival, WBRT may lead to extended damage to the white matter and cause severe disruption of the RSFC in multiple resting-state networks. Apart from well-recognized deficits in memory function, executive functioning and goal-directed attention which is assumed to depend on the interaction of several networks may be severely impaired as a result of WBRT. Although we assume that the patient presented here suffered the typical course of image changes and side effects induced by WBRT, more studies are clearly needed to confirm these findings.

## Data Availability Statement

The raw data supporting the conclusions of this article will be made available by the authors, without undue reservation.

## Ethics Statement

The studies involving human participants were reviewed and approved by Ethics Committees of the Universities of Cologne and Essen, Germany. The patients/participants provided their written informed consent to participate in this study.

## Author Contributions

MK selected, analyzed, and interpreted the data and was a major contributor to writing the manuscript. CL, PL, FM, and NS provided the imaging methods and facilities. MS, GS, and CF treated and examined the patient. CJ and SC developed the network analysis methods and provided the control group data. MK, MR, GF, NG, and K-JL conceptualized the study. All authors read and approved the final manuscript.

## Funding

The Wilhelm-Sander Stiftung, Germany, supported this work. This project was partially funded by the German National Cohort and the 1000BRAINS-Study of the Institute of Neuroscience and Medicine, Research Center Juelich, Germany and received funding from the European Union's Horizon 2020 Research and Innovation Programme under Grant Agreement No. 945539 (HBP SGA3; SC) as well as from the Initiative and Networking Fund of the Helmholtz Association (SC).

## Conflict of Interest

The authors declare that the research was conducted in the absence of any commercial or financial relationships that could be construed as a potential conflict of interest.

## Publisher's Note

All claims expressed in this article are solely those of the authors and do not necessarily represent those of their affiliated organizations, or those of the publisher, the editors and the reviewers. Any product that may be evaluated in this article, or claim that may be made by its manufacturer, is not guaranteed or endorsed by the publisher.
