# Supplementary material for: Case Report: Disruption of Resting-State Networks and Cognitive Deficits After Whole Brain Irradiation for Singular Brain Metastasis
Source: Front Neurosci. 2021 Oct 27;15:738708. doi: 10.3389/fnins.2021.738708 (PMC8578854; doi:10.3389/fnins.2021.738708)
Supplement: Supplementary file 1 [file Table_1.DOCX]

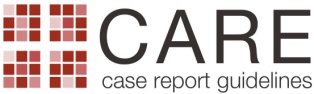
CARE Checklist of information to include when writing a case report
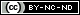


**Topic Item Checklist item description Reported on Line**

**Title 1** The diagnosis or intervention of primary focus followed by the words “case report” P1, line 1

**Key Words 2** 2 to 5 key words that identify diagnoses or interventions in this case report, including "case report" P2, line 36-36

Abstract

**(no references)**

**3a** Introduction: What is unique about this case and what does it add to the scientific literature? P2, line 4-5

**3b** Main symptoms and/or important clinical findings P2, line 17-23

**3c** The main diagnoses, therapeutic interventions, and outcomes P2, line 8-10

**3d** Conclusion—What is the main “take-away” lesson(s) from this case? P2, line 29-34

**Introduction 4** One or two paragraphs summarizing why this case is unique (**may include references**) P3, line 25-32

**Patient Information 5a** De-identified patient specific information P3, line 37-45

**5b** Primary concerns and symptoms of the patient P3, line 38

**5c** Medical, family, and psycho-social history including relevant genetic information P3, line 37

**5d** Relevant past interventions with outcomes P3, 39-46 , P13, Tab. 1

Clinical Findings

**Timeline**

**Diagnostic Assessment**

**Therapeutic Intervention**

**Follow-up and Outcomes**

1. Describe significant physical examination (PE) and important clinical findings P5, line 40-43
2. Historical and current information from this episode of care organized as a timeline P13, Tab. 1

**8a** Diagnostic testing (such as PE, laboratory testing, imaging, surveys). P6, line 1-29

**8b** Diagnostic challenges (such as access to testing, financial, or cultural) not applicable

**8c** Diagnosis (including other diagnoses considered) P3, line 38-39

**8d** Prognosis (such as staging in oncology) where applicable P13, Tab. 1

**9a** Types of therapeutic intervention (such as pharmacologic, surgical, preventive, self-care) P13, Tab. 1

**9b** Administration of therapeutic intervention (such as dosage, strength, duration) P13, Tab. 1

**9c** Changes in therapeutic intervention (with rationale) not applicable

**10a** Clinician and patient-assessed outcomes (if available) P5, line 38 to P6, line 16

**10b** Important follow-up diagnostic and other test results P6, line 18-29

**10c** Intervention adherence and tolerability (How was this assessed?) P13, Tab. 1

**10d** Adverse and unanticipated events P6, 10-16

**Discussion 11a** A scientific discussion of the strengths AND limitations associated with this case report P7, line 1 to P8, line 2

**11b** Discussion of the relevant medical literature **with references** P7, line 1 to P8, line 2

**11c** The scientific rationale for any conclusions (including assessment of possible causes) P7, line 14-21

**11d** The primary “take-away” lessons of this case report (without references) in a one paragraph conclusion P8, line 13-18

**Patient Perspective 12** The patient should share their perspective in one to two paragraphs on the treatment(s) they received P8, line 5-10

**Informed Consent 13** Did the patient give informed consent? Please provide if requested . . . . . . . . . . . . . . . . . . . . . . . . . . . . . . . . . . . . . . **Yes x No**
